# Factors related to technical management and adverse drug event reporting in independent retail pharmacies in Cali, Colombia

**DOI:** 10.1016/j.heliyon.2022.e09016

**Published:** 2022-02-25

**Authors:** Jobany Castro Espinosa, Ángela María Jiménez Urrego, Alejandro Botero Carvajal

**Affiliations:** a Faculty of Health, Universidad Santiago de Cali, Cali, Colombia; b Psychology Program, Faculty of Human and Social Sciences, Universidad de San Buenaventura Cali, Cali, Colombia; c Psychology Program, School of Health, Universidad Santiago de Cali, Cali, Colombia

**Keywords:** Pharmacies, Technical manager, Side effects and adverse drug reactions, Drug effects, Side effects, Cross-sectional studies

## Abstract

Independent retail pharmacies are required to have a technical manager responsible for the operation and adverse drug event reporting. In this context the following investigation is developed based on one objective: To establish factors related to the technical management and adverse drug event reporting in independent retail pharmacies in Cali. This is a cross-sectional observational study. Data was randomly collected from an estimated sample of 244 independent retail pharmacies. The results show that storage was the area of greatest implementation, 94% of the assessed pharmacies had a technical manager and 50% of them reported adverse drug events. A technical manager, being a chemist, pharmacist, or pharmacy manager, was associated with having computer equipment and dispensing homeopathic products. Adverse drug event reporting was directly associated with having bibliographic resources and inversely associated with the technical manager being a drug retailer. These data show the factors related to technical management of independent retail pharmacies and adverse drug event reporting were identified.

## 1 Introduction

Pharmacies are legally permitted to operate in Colombia [1] either as wholesalers or retailers based on whether they sell as wholesale distributors to other stores or operate at the retail level for the general public. Independent retail pharmacies (IRPs) include pharmacies and drugstores [2], which receive and store, distribute, transport, and dispense drugs and medical devices. In addition, they offer injection and blood sugar monitoring services and sell allopathic, homeopathic, and phytotherapeutic products; dietary supplements; cosmetics; toiletries; and sanitary products.

IRPs have become a major player in national public health because people can access medicines through them easily and relatively free of many restrictions. These establishments have an impact on the mass market of medicines [3, 4] and other products [5]. In this regard, self-medication practices recommended by IRP sales people [6] have been considered a public health risk because the staff at these establishments neither have the experience nor the knowledge required to effectively advise users [7]. Colombian regulations acknowledge different levels of professionals involved in this service, including pharmaceutical chemist, pharmacy technologist, drug retailer, drug distributor, and licensed pharmacist. The law requires pharmacies to be represented by a technical manager (TM) who is responsible for the implementation of quality processes in the receipt, storage, and dispensation of medicines, including infrastructure, documentation, and provision of equipment and supplies. TMs are also responsible for providing patients with information on pharmacotherapy, health promotion, and disease prevention as well as the implementation of pharmacovigilance programs [2].

It is vital that these establishments assess the quality of the products they sell, considering that these are for mass consumption and are easily accessed by the community [8]. In addition, IRPs provide controlled drugs [9] and high-risk medications [10], which require a practitioner's prescription [11]. The National Food and Drug Surveillance Institute (INVIMA, for its Spanish acronym), in line with international standards in all countries, frequently issues health alerts regarding the sale of fraudulent or counterfeit products, most of which include drugs that can be purchased in these establishments. Nonetheless, several authors indicate that the reporting of adverse drug events (ADEs) by community pharmacists is low because of diverse barriers and limitations [12, 13].

Considering the increasing number of pharmacies and their impact on public health, it is imperative that pharmacies comply with the legal requirements to operate. Such compliance may, in turn, largely depend on the management by the TM. Furthermore, it is essential to identify the number of pharmacies that report ADEs to ensure the safe use of drugs. Therefore, this study aimed to determine the factors related to the technical management and ADE reporting by IRPs in Santiago de Cali.

## 2 Materials and methods

**Figure 1:**
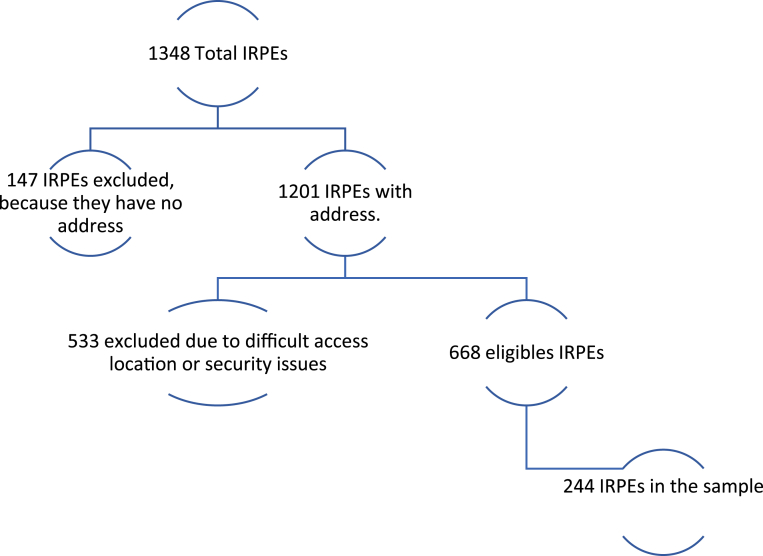
Flowchart of the process of sample size determination.

The present cross-sectional observational study was conducted in Santiago de Cali in 2019. Santiago de Cali is one of Colombia's main cities, located in the southwest of the country, and is divided into 22 administrative units known as *comunas*. The study population comprised IRPs authorized to operate. The list of such establishments in Santiago de Cali was provided by the Valle del Cauca Sanitation Implementing Unit (UES, for its Spanish acronym). Of the total 1348 IRPs listed, 147 were excluded from the study for not indicating address information, thus yielding a population of 1201 IRPs for sampling. When locating the address of the EFIM, another 533 were discarded because they were previously identified as being located in sectors of the city that were difficult to access or had security problems, leaving 668 EFIM. The sample size was determined through the proportion formula for finite populations using the following parameters: 95% confidence interval, 5% allowable error, and estimated proportion of 50%; a sample size of 244 was obtained. Figure 1 presents the process used to determine the sample size.

### 2.1 Data collection process

A simple random sampling method was used to select the establishments to be visited. Based on the list provided by the UES, each IRP was assigned a consecutive number, and 244 establishments were selected through the generation of random numbers, without any substitution. As inclusion criteria, IRPs had to be registered in the UES and the address had to be included in the list. The exclusion criteria were that the pharmacy did not exist at the indicated address, it was closed, or the person-in-charge refused to participate in the study. The data collection method comprised a face-to-face interview as well as a visual inspection checklist and included close-ended and open-ended questions categorized in six sections: 1) establishment identification (*comuna*, legal standing, type and class of establishment, and age of the establishment); 2) human resources (TM, TM's degree, TM's diploma in a visible place, and number of people working in the establishment); 3) infrastructure (areas of the establishment: reception, quarantine, storage, area for medicines subject to special control, area for magistral formulations, administrative department, injection room, and area for dispensing); 4) equipment and supplies (cooling and temperature control equipment, thermal hygrometer, storage shelves, equipment to prepare magistral formulations, fire extinguisher, stowage, computer equipment, equipment to monitor environmental conditions, communication and connectivity, management software, injection room elements, bibliographic resources, glucometer, and blood pressure monitor); 5) ADE reporting (whether ADEs were reported to an entity and the name of that entity. To determine whether ADEs were reported, the actual existence of a report was verified); and 6) type of products sold (allopathic drugs, cosmetics, veterinary drugs, homeopathic products, drugs used for medicinal preparations, phytotherapy products, controlled drugs, medical devices, dietary supplements, and magistral formulations).

To collect the information, the research interviewers went to each establishment and contacted the person-in-charge to present the study. In case of acceptance to participate, the person was asked to sign the informed consent form and then face-to-face interviews were conducted. Refusal to participate resulted in exclusion of the establishment from the study.

### 2.2 Analyses

The data gathered was entered into a Microsoft Excel template and subsequently recoded and exported to Stata 14 software. Using this information, proportions were calculated for qualitative variables with 95% confidence interval, and average and standard deviation values were calculated for quantitative variables. Whether a TM worked at the IRP was determined and if so, their degree was identified. The factors associated with the TM being a pharmaceutical chemist or pharmacy technologist were evaluated. Therefore, the odds ratio (OR) was estimated as a measure of association and its statistical significance was defined through its 95% confidence interval and *P-*value of the Chi-square test. The variables that showed a statistically significant association in the bivariate analysis were included in a multivariate model by multiple logistic regression, choosing the model in which all variables were associated in a statistically significant manner, and the final model also needed to be statistically significant (*P* < 0.05).

According to the provisions of Resolution 8430 of 1993 of the Colombian Ministry of Health, this is considered a risk-free study. Nevertheless, the participants were asked to sign an informed consent. The project was approved by the ethics committee of the Santiago de Cali University.

## 3 Results

Off all the IRPs visited, 10 refused to participate in the study, resulting in a response rate of 96% (244/254). The participants were distributed among 14 of the 22 *comuna*s in the city. The highest number of participating IRPs (27 establishments) belonged to *comuna* No. 2, whereas the lowest participation was observed in *comuna* No. 19 (1 establishment). In terms of legal standing, most of them had legal status (54%), provided commercial services (95%), were drugstores (93%), were single establishments (52%), and had been established since >10 years (48%) (Table 1).

**Table 1:** Description of the participating IRPs.

Variable	If equipped with:	Percentage	CI 95%
**TECHNICAL MANAGER**			
Yes	231	94,7%	91,0%-6,9%
No	13	5,3%	3,1% - 9,0%
**TECHNICAL MANAGER’S DEGREE OR CERTIFICATE**			
Pharmaceutical chemist	7	3,0%	1,4% - 6,2%
Pharmacy technologist	127	54,3%	47,8%-60,6%
Drug dispenser	83	35,5%	29,3%-41,6%
Licensed pharmacist	17	7,3%	4,5% - 11,4%
**LEGAL STANDING**∗			
Legal	132	68,8%	61,8% - 75,0
Mixed	15	7,8%	4,7% - 12,6%
Natural	45	23,4%	17,9%-30,0%
**SERVICE PROVIDED:**			
Health Promoting Entity	8	3,3%	1,6% - 6,4%
Service Provider Institution	3	1,2%	0,4% - 3,8%
Commercial	233	95,5%	92,0%-97,5%
**ESTABLISHMENT TYPE**			
Drugstore	227	93,0%	89,0%-95,6%
Pharmacy	17	7,0%	4,4% - 10,9%
**ESTABLISHMENT CLASS**			
Single	128	52,5%	46,1%-58,7%
Chain	115	47,1%	40,9%-53,4%
Self-service	1	0,4%	0,0% -2,9%
**AGE OF ESTABLISHMENT**∗∗			
Less than 1 year	15	6,2%	3,8% - 10,1%
1–5 years	63	26,1%	20,9%-32,1%
5–10 years	46	19,1%	14,6%-24,6%
More than 10 years	117	48,5%	42,2%-54,9%

∗ The percentage for this option was calculated based on a total of 192 IRPs because 52 of them did not respond to the question.

∗∗ The percentage for this option was calculated based on a total of 241 IRPs because 3 of them did not respond to the question.

The areas most frequently observed were storage and dispensing (98% each), storage shelves were the most frequently used resource (99%), and drugs were most frequently used for compounded preparations (96%) (Table 2). Twenty-eight IRPs dispensed controlled drugs, and 82% of these establishments had the required area to that end. Sixteen IRPs sold magistral formulations, and all of them had the special area to do so. A total of 184 IRPs had an injection room with the corresponding equipment and supplies.

**Table 2:** Description of the infrastructure, equipment and supplies, and products sold by the participating IRPs.

Variable	Equipped with:	Percentage	CI 95%
**Infrastructure**			
Reception	236	96,7%	93,5% a 98,4%
Quarantine area	232	95,1%	91,5% a 97,2%
Storage area	240	98,4%	95,7% a 99,4%
Medicines subject to special control	23	9,4%	6,3% a 13,8%
Magistral formulations	16	6,6%	4,0% a 10,5%
Administrative department	233	95,5%	92,0% a 97,5%
Injection room	184	75,4%	69,6% a 80,4%
Dispensing area	241	98,8%	96,2% a 99,6%
**Equipment and supplies**			
Cooling equipment and thermometer	203	83,2%	77,9% a 87,4%
Thermal hygrometer	225	92,2%	88,1% a 95,0%
Storage shelves or modules	242	99,2%	96,7% a 99,8%
Fire extinguisher	228	93,4%	89,5% a 96,0%
Stowage	126	51,6%	45,3% a 57,9%
Computer equipment and office	201	82,4%	77,0% a 86,7%
Equipment to monitor environmental conditions	225	92,2%	88,1% a 95,0%
Communication and connectivity	225	92,2%	88,1% a 95,0%
Management software	169	69,3%	63,1% a 74,8%
Injection room elements and equipment	184	75,4%	69,6% a 80,4%
Bibliographic resources on pharmaceuticals and related products∗	204	84,0%	78,7% a 88,1%
Glucometer∗	81	33,3%	27,6% a 39,5%
Blood pressure monitor∗	94	38,7%	32.7% a 45,0%
**Products sold**			
Allopathic drugs∗	173	70,6%	65,1% a 76,6%
Cosmetics∗∗	223	91,0%	88% a 94,9
Veterinary drugs∗	4	1,6%	0,6% a 4,3%
Homeopathic products∗	172	70,2%	64,7% a 76,2%
Drugs used for medicinal preparations∗	234	95,5%	93,0% a 98,1%
Phytotherapy products∗	143	58,4%	52,5% a 64,9%
Medicines subject to special control∗	28	11,4%	8,0% a 16,2%
Medical devices∗	162	66,1%	60,4% a 72,3%
Dietary supplements∗	223	91,0%	87,5% a 94,6%
Magistral formulations∗	16	6,5%	4,0% a 10,5%

∗ This option was calculated based on a total of 243 IRPs because 1 of them did not respond to the question.

∗∗ This option was calculated based on a total of 242 IRPs because 2 of them did not respond to the question.

Regarding technical management, most IRPs had a TM (94%) whose diploma was displayed in a visible place (93%). TMs were pharmacy technologists or pharmaceutical chemists in 54.3% of the IRPs. These degrees were directly associated with having an area for controlled drugs; an area for magistral formulations; cooling and temperature control equipment; stowage; computing equipment and management software; handling of homeopathic and phytotherapy products; medical devices; and magistral formulations.

Conversely, this same variable was inversely associated with the establishment being a drugstore, having a blood pressure monitor, and an injection room (Table 3). The average number of workers per drugstore was 3 (standard deviation = 3.0).

**Table 3:** Bivariate analysis.

Dependent variable: The Technical Manager being a pharmaceutical chemist or pharmacy technologist			
Independent variable	OR	CI 95%	*P*-value
Area for medicines subject to special control	5.3	1.5 a 28.8	0.0037
Type (drugstore)	0.2	0.0 a 0.7	0.0074
Area for magistral formulations	11.5	1.7 a 493.1	0.0035
Injection room	0.3	0.1 a 0.6	0.0002
Cooling equipment and thermometer	2.7	1.2 a 6.0	0.0054
Stowage	2.5	1.4 a 4.3	0.0008
Computing equipment	4.8	2.2 a 11.2	0.0000
Management software	3.4	1.8 a 6.4	0.0000
Blood pressure monitor	0.5	0.3 a 1.0	0.0277
Homeopathic products	1.9	1.0 a 3.5	0.0274
Phytotherapy products	2.5	1.4 a 4.5	0.0007
Controlled drugs	7.0	2.0 a 37.1	0.0005
Medical devices	2.0	1.1 a 3.6	0.0138
Magistral formulations	5.7	1.2 a 52.2	0.0119
**Dependent variable: The IRP reporting ADEs**			
**Independent variable**	**OR**	**CI 95%**	***P*-value**
Communication	4.2	1.3 a 17.7	0.0081
Bibliographic resources	4.9	2.1 a 12.9	0.0001
Phytotherapy products	2.0	1.2 a 3.5	0.0078
Controlled drugs	3.4	1.3 a 9.8	0.0053
Medical devices	3.4	2.1 a 7.4	0.0000
Magistral formulations	7.7	1.7 a 71.0	0.0020
Drug dispenser's license	0.5	0.3 a 0.9	0.0084

Only half of the IRPs stated that they report ADEs; of these, 54% report to the Health Department, 26% to the INVIMA, and the remainder did not indicate to whom they report the information. In relation to the reporting of ADEs, the variables associated in a statistically significant manner were having computing equipment; bibliographic resources; dispensation of phytotherapy products and controlled drugs; medical devices; and magistral formulations, whereas, in the opposite case, TMs were drug salesmen (Table 3).

Multiple logistic regression analysis with the TM being a pharmaceutical chemist or pharmacy technologist as the dependent variable identified a model that included the following variables: injection room (OR = 0.31; CI 95% = 0.15–0.64, *P* = 0.002), computing equipment (OR = 3.96; CI 95% = 1.83–8.59; *P* < 0.0000), and dispensation of homeopathic products (OR = 1.97; CI 95% = 1.06–3.64, *P* = 0.031). For the model with ADE reporting as the dependent variable, the variables included were bibliographic resources (OR = 5.45; CI 95% = 2.27–13.08, *P* < 0.000) and the TM being a drug retailer (OR = 0.47; CI 95% = 0.27–0.86, *P* = 0.013) (Table 4). Estimates for both models were correct based on the validation results obtained through the verisimilitude ratio test, adjusted McFadden, adjusted R2, Bayesian Information Criterion (BIC), the Hosmer–Lemeshow test, and the percent correct classification, indicating that both models were suitable (Table 5). The correlation matrix of the independent variables for both models showed correlation coefficient values of <0.8, which indicates that these variables were not correlated, and thus there was no multicollinearity [model 1: having an injection room and computing equipment (correlation = 0.15), having an injection room and selling homeopathic products (correlation = 0.02), having computing equipment and selling homeopathic products (correlation = 0.06); model 2: having bibliographic resources and the TM being a drug retailer (correlation = −0.06)].

**Table 4:** Multiple logistic regression models.

Dependent variable: The Technical Manager being a pharmaceutical chemist or pharmacy technologist∗			
Variable	OR	CI 95%	*P*-value
Injection room	0.3	0.1 a 0.6	0.0020
Computing equipment	4.0	1.8 a 8.6	0.0000
Homeopathic products	2.0	1.1 a 3.6	0.0310
Constant	0.7	0.2 a 1.9	0.4570
**Dependent variable: The IRP reporting ADEs**∗∗			
**Variable**	**OR**	**CI 95%**	***P*-value**
Bibliographic resources	5.4	2.3 a 13.1	0.000
Drug dispenser's license	0.5	0.3 a 0.9	0.013
Constant	0.3	0.1 a 0.7	0.004

∗ Model parameters: *P*-value < 0.000, pseudo R2 = 0.104.

∗∗ Model parameters: *P*-value < 0.000, pseudo R2 = 0.077.

**Table 5:** Model validation.

Validation indicators	Model 1. The Technical Manager being a pharmaceutical chemist or pharmacy technologist	Model 2. The IRP reporting ADEs
LR test (verisimilitude ratio test)	0.00	0.00
Adjusted McFadden	0.08	0.06
Adjusted count R2	0.22	0.24
BIC	306.38	314.40
Hosmer–Lemeshow test	0.88	0.41
Sensitivity	79.85%	69.57%
Specificity	49.49%	55.93%
Correct classification	66.95%	62.66%

## 4 Discussion

In general, the IRPs had most of the surveyed areas. Nonetheless, rather than establishing whether these areas are present, it is vital to assess the degree of compliance with the operational guidelines [14, 15]. Storage areas and environmental conditions may directly or indirectly affect the quality of drugs. Inadequate conditions may pose a public health risk owing to the potential to cause adverse drug reactions [16]. Most IRPs had shelves for the correct arrangement of medicines and medical devices, thus contributing to the correct classification and identification. Proper waste disposal is an important factor because community members may be unfamiliar with this procedure [17]. In this regard, it was found that most IRPs included a quarantine area.

Drug storage facilities must have systems to ensure the temperature and relative humidity conditions recommended by the manufacturer, including the use of thermometers and thermal hygrometers [2]. Drugs may undergo degradation if these conditions are not maintained within the established parameters [16]. Most IRPs in this study were equipped with a cooling device, a thermometer, and a thermal hygrometer. In contrast, a study conducted in Florida, Colombia, showed that none of the studied drugstores had thermal hygrometers, and only a few of them had thermometers [15].

A study conducted by Cohen et al. in a hospital pharmacy found that 89 out of 189 drugs for refrigerated storage were refrigerated [18]. The chain of use of medicines can cause the temperature to be outside the manufacturers' specifications, especially in hot cities such as Santiago de Cali in Colombia. An investigation by Ziance et al. concluded that the transit, handling and storage of pharmaceutical products can expose them to temperatures higher or lower than those recommended by the manufacturer. Therefore, pharmacists must receive, handle and store at controlled temperatures to ensure the dispensing of effective and quality products to patients [19].

In accordance with previous studies [15, 20], we have identified establishments that dispense medicines subject to special control. Although most of those IRPs had the required area for controlled drugs, it is noteworthy that others did not, thus failing to comply with the regulations [21]. It is important to understand the need for strict management of these drugs because they pose a risk of producing dependence and of being traded illegally. The association observed between these establishments and the fact that their TMs were pharmacy technologists or pharmaceutical chemists represents a positive finding for the prevention of such risks. However, it should not be disregarded that two IRPs proposed a drug dispenser as their TM, and one of them even claimed not to have a TM in charge. A study conducted on the management of controlled drugs in the IRPs of Medellin, Colombia, showed that 55.7% of the TMs were drug dispensers, a role that is not the most suitable for this activity according to the authors [9].

Consistent with other studies [14], 75% of the establishments had an injection room. Intravenous administration of drugs or the performance of sensitivity tests is not permitted in these facilities, and a medical prescription should be requested for intramuscular administration of drugs. Injectable drugs may only be administered by authorized personnel who have received the appropriate educational training to do so. This is essential owing to the risks associated with inappropriate administration by this route [22] and the probability of inflicting pain in the application with worse complications [23].

The training and qualification of the IRP personnel were not verified in this study. Therefore, considering the significant number of IRPs that provide this service and the potential risks involved, we suggest that further studies be conducted to analyze this aspect. In cases where the service of blood sugar monitoring by finger-prick devices is offered, it is imperative that the TM be a pharmaceutical chemist or pharmacy technologist. However, we identified an inverse association between having a pharmaceutical chemist or pharmacy technologist as a TM and having an injection room; therefore, it is statistically unlikely that IRPs that include this area will have a pharmaceutical chemist or pharmacy technologist as a TM.

An association was found between dispensing homeopathic products and the TM being a pharmaceutical chemist or pharmacy technologist. Some customers may prefer the use of these products as a therapeutic alternative for their health care. It is important for pharmacists to be familiar with the principles on which these products are based to accurately advise customers regarding the use. Moreover, it is vital to reinforce knowledge about the use of homeopathic products during professional training because several universities in Colombia do not include it in their study plan [21].

Although homeopathic medicine has some controversy about its effectiveness, some studies try to explain how it acts in the organism. Considering that these drugs are sold in EFIM, it is important, in addition to their delivery, the advice that a pharmacist provides to those who use them [24]. The lack of knowledge about the safety of the raw materials used in their preparation deserves to be reviewed, because more and more of these drugs are used by the common population [25]. A systematic review by Ernst et al. concluded that the available evidence for homeopathy does not justify the recommendation of its use in clinical practice [26].

Of all the IRPs visited, only 17 (7%) were pharmacies. Unlike regular drugstores, pharmacies prepare magistral formulations [1] and therefore have to comply with special requirements [22]. It was found that all the establishments that dispense magistral formulations had a specific area for such purpose. Consistent with other studies [18], it was observed that the establishments had a TM and qualified personnel in charge such as drug dispensers, pharmacy assistants, or pharmacy technologists. It is vital that the staff have the necessary training and experience [7, 14] because many of them advise on the use of medications [14, 23], including some types that they should not recommend [18].

In relation to this, most establishments analyzed had bibliographic resources on pharmaceutical products, in contrast to a study that showed the lack of these references in drugstores on the grounds that their employees did not need them considering their vast knowledge and experience [15]. Considering the constant demands that arise in this field, it is essential that pharmacy personnel receive training on a permanent basis [27].

EFIMs are vital to the public health of a community, as they are very accessible to the population. A systematic review by Eades et al. found that pharmacists' confidence in providing public health services was medium to low and that time commitment, lack of counseling space, lack of demand, and the expectation of negative customer feedback were identified as limiting factors in the provision of these services. Of the consumers, it was found that most had never received public health services from their pharmacist, that they viewed pharmacists as adequate providers of public health counseling, and that satisfaction was high in those who had received pharmaceutical public health [28].

The impact on public health depends on the capabilities of its staff and how people perceive it. A study in five Danish cities identified that pharmacy staff and physicians have a higher expectation of pharmacists' medical knowledge, compared with local politicians, who had a lower expectation. For their part, customers consider pharmacists to be part of the health sector with high knowledge of medicines. While politicians were not aware of the competencies of pharmacy, they were open to the use of pharmacy in the health system [29].

Although pharmacovigilance programs are crucial to drug safety and thus public health, their implementation and operation in EFIMs may not be optimal. A study with 1857 Lebanese community pharmacists showed that the majority had a good knowledge of pharmacovigilance and that most had a positive attitude towards adverse reaction reporting, considering this activity as one of the main activities. However, a lack of practice and training in pharmacovigilance was found [30].

Any establishment can join INVIMA's national pharmacovigilance program and submit reports of ADEs. These programs are usually well implemented in health institutions; the greater their complexity, the more sophisticated the reporting and analysis methods.

However, the implementation of these programs in IRPs is inefficient and in most cases, they do not even work. Considering that most ADEs are likely to occur in the ambulatory population and that because they are not reported, the magnitude of this problem is not evident, this situation represents a great opportunity to train staff on pharmacovigilance. Multivariate analysis showed that the establishments that reported ADEs were directly associated with having bibliographic resources, a valuable tool for the management and analysis of ADEs. Conversely, it was inversely associated with the TM being certified as a drug dispenser, which reveals that these personnel are not involved in the reporting process on a regular basis.

Regarding the limitations of the study, it was not possible to thoroughly assess compliance in some cases because they were not readily visible, for example, some establishments claiming to report ADEs did not show such reports. This may indicate that several shortcomings identified in the study could actually be worse.

Based on the results of the present study, we suggest that government agencies verify the correct operation of these establishments, including that they have the areas mandatory by law and that they fulfill the requirement of having a TM. In case of non-compliance, they should be accordingly informed. For pharmacies that dispense magistral formulations, it is important to note that the TM should be a pharmaceutical chemist or pharmacy technologist.

## 5 Conclusion

An association was found between having a TM who is a pharmaceutical chemist or pharmacy technologist and having computer equipment, dispensing homeopathic products, and not offering injection services. Finally, an association was observed between reporting ADEs and having bibliographic resources in the facilities as well as not having a pharmacy technologist working as a TM. Colombian regulations require that EFIMs be under the technical direction of qualified professionals; the results of this study established the importance of this personnel, in the sense that their presence allows them to function properly. EFIMs have an important impact on the public health of a region, since they are a source for the acquisition of drugs with or without a medical prescription, thus influencing rational use and self-medication practices. The fact that these establishments are staffed by qualified personnel is crucial, in the sense that they provide adequate guidance to consumers. In this way, for pharmacovigilance and the reporting of adverse drug events, it is essential to have elements such as bibliographic resources, personnel and their training so that it can be carried out adequately.

## Declarations

### Author contribution statement

Angela Jimenez: Wrote the paper analyzed and interpreted the data.

Jobany castro: Conceived and designed the experiments analyzed; Analyzed and interpreted the data.

Alejandro botero: Analyzed and interpreted the data; Wrote the paper.

### Funding statement

This work was supported by Dirección General de Investigaciones of Universidad Santiago de Cali under call No. 01-2021.

### Data availability statement

Data will be made available on request.

### Declaration of interests statement

The authors declare no conflict of interest.

### Additional information

No additional information is available for this paper.
